# Protopanaxadiol, an Active Ginseng Metabolite, Significantly Enhances the Effects of Fluorouracil on Colon Cancer

**DOI:** 10.3390/nu7020799

**Published:** 2015-01-23

**Authors:** Chong-Zhi Wang, Zhiyu Zhang, Jin-Yi Wan, Chun-Feng Zhang, Samantha Anderson, Xin He, Chunhao Yu, Tong-Chuan He, Lian-Wen Qi, Chun-Su Yuan

**Affiliations:** 1 Tang Center for Herbal Medicine Research, University of Chicago, Chicago, IL 60637, USA; E-Mails: czwang@uchicago.edu (C.-Z.W.); zzhang2@bsd.uchicago.edu (Z.Z.); wanjinyi1128@163.com (J.-Y.W.); zhangchunfeng67@163.com (C.-F.Z.); sanderson6@uchicago.edu (S.A.); xhe@163.com (X.H.); yuch@hyit.edu.cn (C.Y.); fleude@126.com (L.-W.Q.); 2 Department of Anesthesia & Critical Care, University of Chicago, Chicago, IL 60637, USA; 3 Department of Orthopedic Surgery, University of Chicago, Chicago, IL 60637, USA; E-Mail: tche@bsd.uchicago.edu; 4 Committee on Clinical Pharmacology and Pharmacogenomics, University of Chicago, Chicago, IL 60637, USA

**Keywords:** protopanaxadiol, ginseng, metabolite, enteric microbiome, fluorouracil, colorectal cancer, HCT-116 cells, cell proliferation, cell cycle, apoptosis, athymic mice, xenograft

## Abstract

In this study, we evaluated the effects of protopanaxadiol (PPD), a gut microbiome induced ginseng metabolite, in increasing the anticancer effects of a chemotherapeutic agent fluorouracil (5-FU) on colorectal cancer. An *in vitro* HCT-116 colorectal cancer cell proliferation test was conducted to observe the effects of PPD, 5-FU and their co-administration and the related mechanisms of action. Then, an *in vivo* xenografted athymic mouse model was used to confirm the *in vitro* data. Our results showed that the human gut microbiome converted ginsenoside compound K to PPD as a metabolite. PPD and 5-FU significantly inhibited HCT-116 cell proliferation in a concentration-dependent manner (both *p* < 0.01), and the effects of 5-FU were very significantly enhanced by combined treatment with PPD (*p* < 0.01). Cell cycle evaluation demonstrated that 5-FU markedly induced the cancer cell S phase arrest, while PPD increased arrest in G1 phase. Compared to the control, 5-FU and PPD increased apoptosis, and their co-administration significantly increased the number of apoptotic cells (*p* < 0.01). Using bioluminescence imaging, *in vivo* data revealed that 5-FU significantly reduced the tumor growth up to Day 20 (*p* < 0.05). PPD and 5-FU co-administration very significantly reduced the tumor size in a dose-related manner (*p* < 0.01 compared to the 5-FU alone). The quantification of the tumor size and weight changes for 43 days supported the *in vivo* imaging data. Our results demonstrated that the co-administration of PPD and 5-FU significantly inhibited the tumor growth, indicating that PPD significantly enhanced the anticancer action of 5-FU, a commonly used chemotherapeutic agent. PPD may have a clinical value in 5-FU’s cancer therapeutics.

## 1. Introduction

Ginseng is a popular herbal medicine worldwide. Asian ginseng and American ginseng are two commonly used ginseng species [1]. It is generally accepted that the major pharmacological constituents of both ginsengs are ginsenosides, a group of steroidal saponins [1,2,3]. Over 80 ginsenosides have been identified, and almost all ginsenosides can be found in these two ginseng species [4,5].

Asian ginseng is one of most studied natural products in different animal models [1,3]. A large case-controlled clinical study in Korea showed that Asian ginseng consumers had a decreased risk for many different cancers compared with those not taking the ginseng regularly [6,7]. Our group has published a number of studies that have demonstrated the chemoprevention potential of American ginseng on colorectal cancer, a major cause of cancer death worldwide. Our data showed, in both *in vitro* and *in vivo* settings, American ginseng extract, fractions, and single compounds possessed significant colon cancer therapeutic potential [8,9,10,11]. Of note, American ginseng has approximately two times higher total ginsenoside content than Asian ginseng [4,5]. The content ratio of protopanaxadiol group ginsenosides (PPDs) *vs.* protopanaxatriol group ginsenosides (PPTs) in American ginseng is approximately 4:1, and thus protopanaxadiol group ginsenosides occupy the majority in the total saponins [9].

Like most other natural products, the route of administration of ginseng is nearly always oral. After ginseng ingestion, its bioavailability is low due to incomplete parent compound absorption and the conversion of parent compounds to metabolites by the intestinal microbiome [12,13,14]. In natural product research, many previous studies have employed primarily reductionist methodologies in parent compound bioactivity screening. However, the bioavailability and bioactivity of their metabolites, an important issue linked to the *in vivo* effects, have often been overlooked. Study of the biotransformation pathways of protopanaxadiol group ginsenosides demonstrated that, via the enteric microbiota, compound K is a major ginseng metabolite with obviously stronger anticancer effects compared to its parent compounds, such as ginsenosides Rb1, Rc and Rd [10,15]. Previous reports have shown that PPD can be converted by gut bacteria from ginseng extract [16] or Rb1 [17]. However, before this study, there was no direct evidence that PPD could be converted from compound K.

Currently, newer chemotherapeutic and chemopreventive agents continue to be investigated, including those derived from botanical sources. Patients with cancer often resort to herbal medicines, including ginseng, to reduce the side effects of chemotherapy or even attempt to increase the effects of the drug treatment [18,19]. A major concern of herb and drug co-administration is their interactions [20,21]. Previous evidence suggested that intestinal microbiota could convert protopanaxadiol group ginseng saponins to PPD. We have shown, using human colorectal cancer cells *in vitro*, that ginseng or ginseng compounds did not reduce, but enhanced the effects of fluorouracil (5-FU), a frequently used chemotherapeutic agent for colon cancer treatment [22,23,24,25]. However, in respect to other active ginseng compounds, such as PPD [11], its interactions with 5-FU have not been evaluated.

In this study, we first demonstrated that compound K could be converted to PPD as a metabolite by the human gut microbiome. Next, we chemically synthesized PPD to obtain enough quantity for the following pharmacological observations. Subsequently, an *in vitro* colorectal cancer cell proliferation test was conducted to evaluate the effects of PPD and 5-FU co-administration and the related mechanisms of action. Finally, an *in vivo* xenografted athymic mouse model was used to confirm the observed *in vitro* data in which PPD significantly enhanced the activities of 5-FU. To verify the validity of the *in vivo* results, the tumor inhibition effects were assessed by three methods, *i.e.*, Xenogen bioluminescence imaging, manual tumor size and tumor weight measurements.

## 2. Materials and Methods

### 2.1. In Vitro Biotransformation

Previously collected stool samples from healthy adult subjects were used [26]. The stool was suspended in saline, and the enteric bacteria fraction was obtained after centrifugation, and mixed with anaerobic medium containing compound K (5 mg/mL) [14]. The mixture was anaerobically incubated at 37 °C for 24 h. Then, the reacted mixtures were processed [14,27]. HPLC/Q-TOF-MS analysis was performed on an Agilent 1290 LC system (Agilent Technologies, Waldbronn, Germany). The separation was carried out on an Agilent Zorbax Extend-C18 UPLC column (4.6 mm × 250 mm, 5 μm) with a constant flow rate of 1 mL/min at 25 °C. The mobile phase was composed of water (0.1% formic acid, A) and acetonitrile (0.1% formic acid, B). Gradient elution started with 20% B and held for 30 min, changed to 45% B for 30 min, changed to 75% B for 18 min, changed to 100% B for 2 min and held for 6 min. The sample volume injected was set at 2 μL. Analysis was performed on an Agilent G6540 Q-TOF-MS spectrometer equipped with an ESI interface. The constituents were analyzed in negative mode. The system was operated under MassHunter workstation software, version B.02.00. Metabolites of the compound K were determined [14].

### 2.2. Preparation of Protopanaxadiol (PPD) and Chemicals Used in this Study

Total ginsenosides (2.0 g), *n*-butanol (250 mL), and sodium hydroxide (10 g) were added to a 500 mL round bottom flask. The mixture was heated to 130 °C and stirred with argon for 2 days and allowed to cool at room temperature. Then, the reaction mixture was washed with water (2 × 100 mL), 1% HCl (2 × 100 mL), 5% NaHCO_3_, and brine. The organic phase was dried over magnesium sulfate. The removal of the solvent under reduced pressure resulted in a sticky oil, which was purified by a silica gel column to release PPD.

The purity of the PPD was determined through HPLC analysis. The HPLC system was a Waters 2960 instrument with a 996 photodiode array detector (Milford, MA, USA). The separation was carried out on a Restek Ultra C8 column (5 μ, 250 × 4.6 mm) (Bellefonte, PA, USA) at 25 °C. Water (solvent A) and acetonitrile (solvent B) were used. Gradient elution started with 60% B, was changed to 95% B for 30 min and held for 2 min. The flow rate was 1.0 mL/min and the detection wavelength was set to 202 nm.

Before observations, the PPD was dissolved in dimethyl sulfoxide (DMSO) to make stock solution and kept at −80 °C as aliquots. Compound K was obtained from ChromaDex Inc. (Irvine, CA, USA). Fluorouracil (5-FU) was obtained from American Pharmaceutical Partners (Schaumburg, IL, USA). Unless specifically indicated, all other chemicals used in this study were obtained from Sigma-Aldrich (St. Louis, MO, USA) or Fisher Scientific (Pittsburgh, PA, USA).

### 2.3. Cell Culture

HCT-116 human colorectal cancer cells (ATCC, Manassas, VA, USA) were routinely grown in a humidified atmosphere of 5% CO_2_ at 37 °C with McCoy’s 5A medium, supplemented with 10% fetal bovine serum and 50 IU penicillin/streptomycin. Cells were grown in a 25-mL flask and were routinely subcultured using 0.05% trypsin-EDTA solution. Cells were maintained at the culture conditions described above for subsequent experiments.

### 2.4. Cell Proliferation Assay

To examine the antiproliferation effect of the study agents, HCT-116 cells were seeded in 96-well plates at approximately 1 × 10^4^ cells/well. After 24 h, indicated concentrations of drugs were added to the wells. After treatment for 48 h, cell proliferation was evaluated using an MTS assay according to the manufacturer’s instructions. Briefly, the medium was replaced with 100 μL of fresh medium and 20 μL of MTS reagent (CellTiter 96 Aqueous Solution, Promega, Madison, WI, USA) in each well, and the plate was returned to the incubator for 1–2 h. A 60 μL aliquot of medium from each well was transferred to an ELISA 96-well plate and its absorbance at 490 nm was recorded.

### 2.5. Cell Cycle Analysis

The HCT-116 cells were plated at a density of 2 × 10^5^ cells onto 24-well plates. The medium was replaced 24 h after seeding with fresh medium containing PPD (10 and 20 μM) and/or 5-FU (30 μM). To analyze the cell cycle distribution, cells were trypsinized after 48 h of exposure to the drugs, fixed gently with 80% ethanol, and stored at −20 °C for 2 h. They were then treated with 0.25% Triton X-100 for 5 min in an ice bath. The cells were resuspended in 300 μL of PBS containing 40 μg/mL propidium iodide (PI) and 0.1 mg/mL RNase. The cells were incubated in a dark room for 20 min at room temperature, and cell cycle analysis was performed using a FACScan flow cytometer (Becton Dickinson, Mountain View, CA, USA) and FlowJo 9.5 software (Tree Star, Ashland, OR, USA). For each measurement, at least 10,000 cells were counted.

### 2.6. Apoptosis Analysis

For apoptosis detection, floating cells in the medium and adherent cells were collected after 48 h of treatment with PPD (10 and 20 μM) and/or 5-FU (30 μM). Using an Annexin V Apoptosis Detection Kit (BD Biosciences, Rockville, MD, USA), cells were stained with annexin V-FITC and propidium iodide (PI) according to the manufacturer’s instructions. Untreated cells were used as the control for double staining. Cells were analyzed immediately using a FACScan flow cytometer. For each measurement, at least 20,000 cells were counted.

### 2.7. In Vivo Antitumor Evaluation

Human colorectal cancer cell line, HCT-116-Luc, was used to establish a xenograft model in immunodeficient BALB/c nude mice. HCT-116-Luc cell line that stably expresses firefly luciferase was generated using a retroviral vector expressing firefly luciferase [28]. Briefly, recombinant retrovirus was packaged in HEK-293 cells by co-transfecting cells with pSEB-Luc and pAmpho packaging plasmid using LipofectAMINE (Invitrogen, Carlsbad, CA, USA). Pooled stable cells (designated HCT-116-Luc) were selected with blasticidin S (0.6 µg/mL) for 7 days. The firefly luciferase activity was confirmed by using Promega’s Luciferase Assay kit (Promega, Madison, WI, USA).

The use and care of animals were carried out under the guidelines approved by the Institutional Animal Care and Use Committee (ACUP number: 70917, approval date: 12 April 2014). Female BALB/c nude mice (4–6 weeks of age, Harlan, Indianapolis, IN, USA) were used. Subconfluent HCT-116-Luc cells were harvested and resuspended in PBS to a density of 2 × 10^7^ cells/mL. Prior to inoculation, cell viability was tested by 0.4% trypan blue exclusion assay (viable cells > 90%). Approximately 1 × 10^6^ HCT-116-Luc cells in 100 μL PBS were injected subcutaneously into both flanks of each animal. The cancer cell inoculation was performed seven days before Day 1. There were four animal groups (*n* = 8/group) in this study, and starting on Day 1 mice received PPD, 5-FU, or vehicle injection intraperitoneally (IP): (1) “5-FU group”, 5-FU 30 mg/kg (a dose lower than the commonly used cancer treatment dose) once a week; (2) “PPD15 + 5-FU group”, PPD 15 mg/kg every other day plus 5-FU 30 mg/kg once a week; (3) “PPD30 + 5-FU group”, PPD 30 mg/kg every other day plus 5-FU 30 mg/kg once a week; (4) “Control group”, vehicle injection.

For Xenogen bioluminescence imaging, animal whole body optical imaging was carried out as previously described [28]. Animals were imaged with a Xenogen IVIS 200 imaging system (Caliper Life Sciences, Hopkinton, MA, USA) at the indicated time points. D-Luciferin sodium salt (Gold Biotechnology, St. Louis, MO, USA) at 150 mg/kg body weight in 0.1 mL sterile PBS was administered IP as a substrate before imaging. Acquired pseudo images were collected by superimposing the emitted light over the grayscale photographs of the animal. Quantitative image analysis was performed with Living Image 4.2 software (Caliper Life Sciences, Hopkinton, MA, USA).

For tumor size calculations, the tumor was measured twice a week. Tumor size in volume (mm^3^) was calculated using the following formula: Tumor volume = (width^2^ × length)/2. For tumor weight measurements, at the end of the observation period, animals were sacrificed and the induced tumors were surgically removed. The tumor weight was immediately measured at room temperature on an electrical balance.

### 2.8. Statistical Analysis

Data are presented as mean ± standard error (SE). *In vitro* and *in vivo* data were analyzed using analysis of variance (ANOVA) for repeated measures and Student’s *t*-test. The level of statistical significance was set at *p* < 0.05.

## 3. Results

### 3.1. Compound K Biotransformation and PPD Purity Analysis

**Figure 1:**
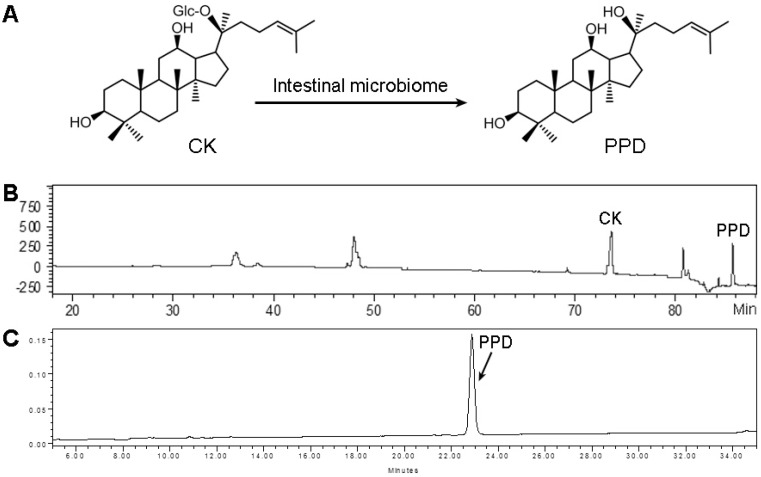
*In vitro* evaluation to assess the effects of the human stool microbiome in metabolizing compound K (CK) and the protopanaxadiol (PPD) purity test. (**A**) Chemical structures of CK and PPD; (**B**) typical HPLC-MS chromatogram of CK biotransformation by the intestinal microbiome; (**C**) the purity of synthesized PPD was determined using HPLC-UV analysis.

After anaerobically incubating compound K with a human stool sample, PPD, as a major metabolite of compound K, was detected via HPLC/Q-TOF-MS analysis (Figure 1B). When the sterilized stool sample was used for the same incubating procedure, there was no PPD detected suggesting the enteric microbiome plays a key role in the observed biotransformation.

To order to obtain a relatively large amount of PPD for subsequent *in vitro* and *in vivo* anticancer pharmacological observations, the PPD was synthesized as described in the Materials and Methods section. The HPLC-determined purity was 95.3% (Figure 1C).

### 3.2. Effects of PPD and 5-FU on HCT-116 Colon Cell Proliferation

**Figure 2:**
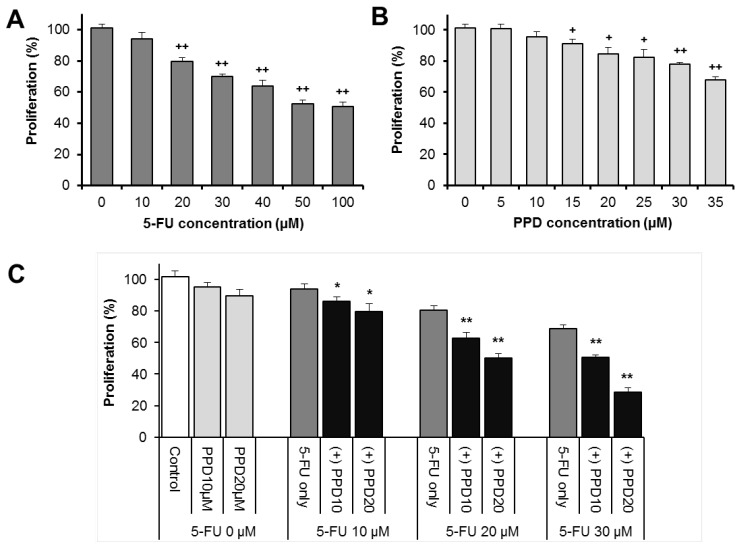
Antiproliferative effects of (**A**) 5-FU and (**B**) protopanaxadiol (PPD) on the HCT-116 human colorectal cancer cells after 48 h of treatment. PPD and 5-FU significantly inhibited the proliferation of the cancer cells; (**C**) when PPD is combined with 5-FU, the antiproliferative effects of 5-FU were significantly enhanced by PPD. ^+^ *p* < 0.05; ^++^ *p* < 0.01 compared to control. * *p* < 0.05, ** *p* < 0.01 compared to 5-FU group.

Figure 2A,B show the antiproliferative effects of 5-FU and PPD on HCT-116 human colorectal cancer cells. After treatment for 48 h, compared with control (normalized to 100%), PPD or 5-FU significantly inhibited HCT-116 cell proliferation in a concentration-dependent manner (both *p* < 0.01).

The influence of PPD on 5-FU induced antiproliferation in the cancer cells is shown in Figure 2C. When used alone for 48 h, 5-FU (10, 20, 30 μM) decreased the cell growth by 5.7 ± 3.9% and 20.2 ± 2.4% and 30.1 ± 1.6%, respectively (*p* > 0.05, *p* < 0.01 and *p* < 0.01 respectively, compared with control). Combined treatment of PPD (10 μM) with 5-FU (10, 20, 30 μM) decreased the HCT-116 cell growth by 13.8 ± 2.6%, 27.4 ± 3.7% and 49.4 ± 1.7%, respectively (*p* < 0.05, *p* < 0.01 and *p* < 0.01 respectively, compared with 5-FU alone). When 5-FU was combined with PPD 20 μM, cell growth was further decreased (Figure 2C). These results suggested that PPD significantly enhanced 5-FU induced antiproliferative effect on the cancer cells.

### 3.3. Effect of PPD and 5-FU on Cell Cycle

**Figure 3:**
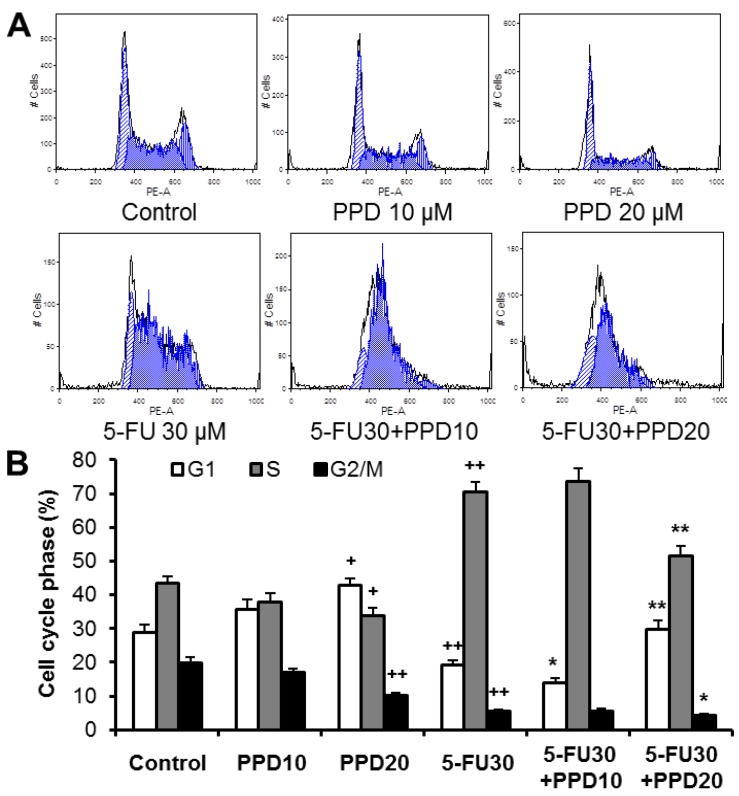
Effects of protopanaxadiol (PPD) on 5-FU-induced cell cycle arrest. HCT-116 human colorectal cancer cells were treated with PPD at various concentrations (10 and 20 µM) in the absence or presence of 5-FU (30 µM) for 48 h. The cell cycle was assessed using PI/RNase staining and flow cytometric analysis. (**A**) The representative histograms of DNA content in each experimental group; (**B**) percentage of each cell cycle phase with various treatments or with control. Data obtained from triplicate experiments. ^+^ *p* < 0.05; ^++^ *p* < 0.01 compared to control; * *p* < 0.05; ** *p* < 0.01 compared to 5-FU group.

In this experiment, we examined whether the decrease of the cancer cell growth is a consequence of the cell cycle arrest at a specific phase. As shown in Figure 3, the cell cycle profile in the control group was G1 28.9%, S 43.3% and G2/M 19.9%. When treated with 30 µM of 5-FU, the cell cycle profile was changed to G1 19.3%, S 70.4% and G2/M 5.7%. 5-FU markedly induced S phase arrest of the cell cycle. On the other hand, treatment with 20 µM of PPD significantly increased cell cycle arrest in the G1 phase. Following exposure to 20 µM of PPD plus 30 µM of 5-FU for 48 h, the percentage of HCT-116 cells at the G1 phase of the cell cycle increased to 29.7%, compared with those exposed only to 5-FU (*p* < 0.01), while the percentage of cells in the S and G2/M phases was significantly reduced with the co-treatment (Figure 3B).

### 3.4. Apoptotic Effects of PPD and 5-FU on HCT-116 Cells

**Figure 4:**
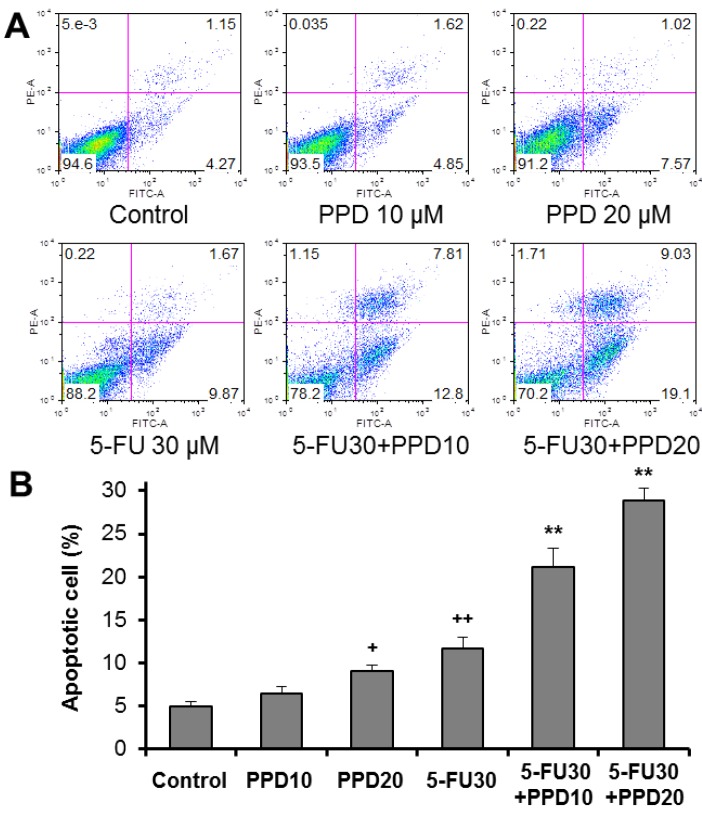
Effects of protopanaxadiol (PPD) on 5-FU-induced apoptosis. HCT-116 human colorectal cancer cells were treated with PPD at various concentrations (10 and 20 µM) in the absence or presence of 5-FU (30 µM) for 48 h. Apoptosis was quantified using annexin V/PI staining followed by flow cytometric analysis. (**A**) Representative scatter plots of PI (*y*-axis) *vs.* annexin V (*x*-axis) in each experimental group; (**B**) the percentage of apoptotic cells. Data obtained from triplicate experiments. ^+^ *p* < 0.05; ^++^ *p* < 0.01 compared to control; ** *p* < 0.01 compared to 5-FU group.

To examine whether the observed cell growth inhibition was caused by apoptosis, using flow cytometry, the induction of apoptosis was determined. The cytograms of bivariate annexin V/PI analysis of the HCT-116 cells are shown in Figure 4A. Compared to the untreated control (apoptosis 5.0 ± 0.6), 10 and 20 µM of PPD increased apoptosis to 6.4 ± 0.9% and 9.1 ± 0.7%, respectively (*p* > 0.05 and *p* < 0.05 respectively, compared with control) (Figure 4B). These data showed that PPD increased the proportion of apoptotic cells at 20 µM. Incubation with 5-FU at 30 μM for 48 h significantly increased apoptotic cells (*p* < 0.01 compared with control). When 5-FU (30 μM) was combined with PPD (10 or 20 µM), the percentage of apoptotic cells increased to 21.1 ± 2.2% and 28.8 ± 1.5%, respectively, which are significantly higher compared with values of the 5-FU treatment alone (both *p* < 0.01). These results suggested that the cell growth inhibition of combined PPD and 5-FU was related to the apoptotic induction (Figure 4).

### 3.5. Effects of PPD and 5-FU on Colon Tumor Growth Inhibition using Xenogen Bioluminescence Imaging

**Figure 5:**
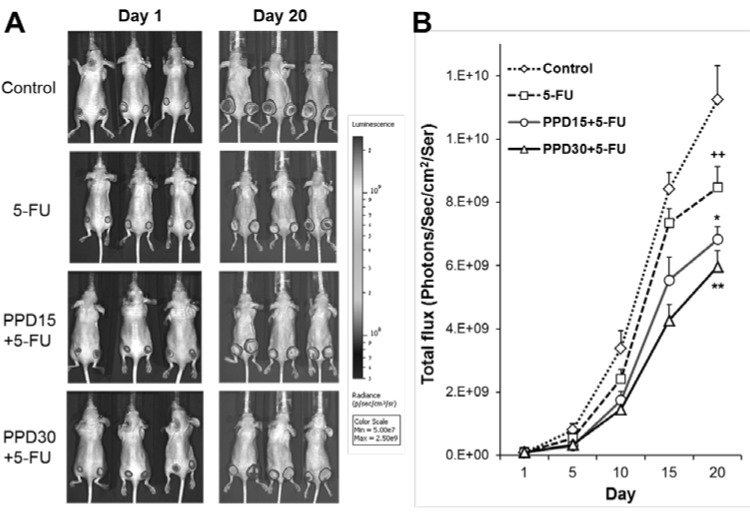
Protopanaxadiol (PPD) enhances the antitumor effects of 5-FU in xenografted athymic mice measured by Xenogen bioluminescence imaging. The tumor growth is monitored from Day 1 (7 days after human colon cancer cell HCT-116 inoculation) to Day 20. (**A**) Representative Xenogen imaging results from animals in different treatment groups; (**B**) quantitative analysis of Xenogen bioluminescence imaging data. Average tumor sizes at the indicated time points are expressed with imaging signal intensities (photons/second/cm^2^/ser). *n* = 8/group. 5-FU, 5-FU 30 mg/kg; PPD15 + 5-FU, PPD 15 mg/kg plus 5-FU 30 mg/kg; PPD30 + 5-FU, PPD 30 mg/kg plus 5-FU 30 mg/kg. ^++^ *p* < 0.01 compared to the model group; * *p* < 0.05; ** *p* < 0.01 compared to the 5-FU group.

Figure 5A shows the representative Xenogen imaging results from animals in different treatment groups. Figure 5B shows the quantitative analysis of Xenogen bioluminescence imaging data on Day 1 and Day 20. Average tumor size at the indicated time points is expressed by imaging signal intensities (photons/second/cm^2^/ser).

Using bioluminescence imaging, the data showed that compared to the control, the 5-FU group significantly reduced the tumor growth (*p* < 0.05). The PPD15 plus 5-FU and PPD30 plus 5-FU groups significantly reduced the tumor size in a dose-related manner (*p* < 0.05 and *p* < 0.01, respectively compared to the 5-FU group), indicating that PPD enhanced 5-FU’s antitumor effects.

### 3.6. Effects of PPD and 5-FU on Colon Tumor Size and Tumor Weight Changes

**Figure 6:**
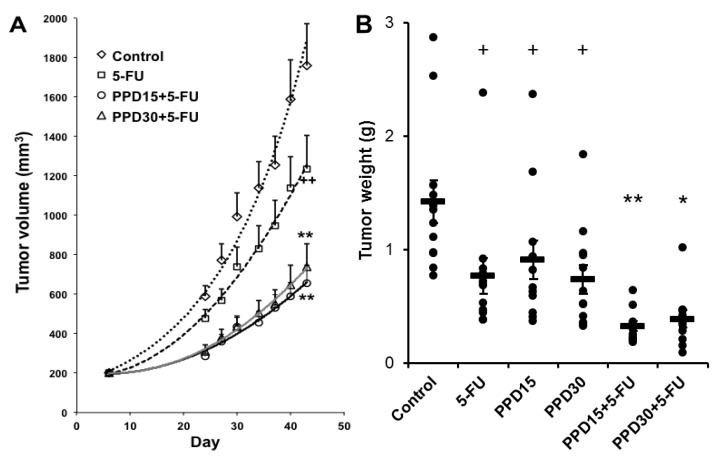
Protopanaxadiol (PPD) enhances the antitumor effects of 5-FU in xenografted athymic mice measured by tumor size and tumor weight. The tumor growth is monitored up to Day 43. (**A**) Changes in tumor size (in volume); (**B**) changes in tumor weight. At Day 43, tumor weights are presented as a scatter plot with mean values and error bars. 5-FU, 5-FU 30 mg/kg; PPD15, PPD 15 mg/kg; PPD30, PPD 30 mg/kg; PPD15 + 5-FU, PPD 15 mg/kg plus 5-FU 30 mg/kg; PPD30 + 5-FU, PPD 30 mg/kg plus 5-FU 30 mg/kg. *n* = 8/group. ^+^ *p* < 0.05; ^++^ *p* < 0.01 compared to the model group; * *p* < 0.05; ** *p* < 0.01, compared to the 5-FU group.

Figure 6A shows the tumor size (in volume) changes with different treatments. The tumor growth was monitored up to Day 43. The tumor size data in different groups, up to Day 20, is comparable to the data obtained from the bioluminescence imaging. PPD15 plus 5-FU and PPD30 plus 5-FU groups very significantly reduced the tumor size (both *p* < 0.01 compared to the 5-FU group). However, the dose-related effect of PPD plus 5-FU was not evident.

Figure 6B shows the tumor weight changes in different treatment groups monitored at Day 43. The trend of tumor weight data is comparable to the data from the bioluminescence imaging and tumor size results. The PPD15 plus 5-FU and PPD30 plus 5-FU groups significantly reduced the tumor weight (*p* < 0.01 and *p* < 0.05, respectively, compared to the 5-FU group). However, similar to the tumor size data, no dose-related effects were seen from the PPD plus 5-FU groups.

## 4. Discussion

Colorectal cancer is one of the most common malignancies and a major cause of cancer death in both men and women worldwide [29]. Although early diagnosis with rigorous screening may have reduced the incidence of this cancer compared to that of several years ago, the prognosis associated with metastatic disease continues to remain bleak [30]. The low 5-year survival rate using the currently available chemotherapy underscores the fact that better anticancer drugs need to be developed. Based on the fact that many oncology drugs have been developed from botanical sources, there is a significant untapped resource in herbal medicines, which needs to be investigated against cancers in the gastrointestinal system [19,31,32,33]. In the last 10 years, we have published many studies using ginseng compounds alone or as adjuncts to existing chemotherapy to improve efficacy and reduce chemotherapeutic agent-induced adverse events [4,10,24,25].

The enteric microbiome plays an important role subsequent to oral American ginseng administration in converting ginseng parent compounds to their metabolites [4,12,34,35]. Study data showed that compound K is a major metabolite that reaches systemic circulation, and this metabolite likely possesses significant anticancer activities [10,11,36,37]. Previously published data revealed that PPD was converted by intestinal microbiota either from total ginseng extract [16] or Rb1 [17]. However, there was no direct evidence that PPD can be converted from compound K. We recently reported the conversion of ginseng saponins by the human enteric microbiome and detected over 20 metabolites [14]. In this study, we showed, for the first time, that using the human enteric microbiome compound K was converted to its metabolite—protopanaxadiol (PPD)—and we also showed PPD is an important bioactive metabolite.

Fluorouracil (5-FU) is one of the most widely used chemotherapeutic agents in first-line therapy for colorectal cancer [22,23], and an overall survival benefit after fluorouracil-based chemotherapy has been established [38]. For the treatment of metastatic colon cancer, however, higher 5-FU doses produced more adverse events while possibly not being more effective than lower doses [39]. If combining 5-FU with other agents can decrease the dose of 5-FU while increasing its anticancer effect, the patient may benefit from the new treatment regimen. However, 5-FU in combination with ginseng compounds has seldom been investigated in *in vivo* settings [21,25].

In this study, an *in vitro* HCT-116 colorectal cancer cell proliferation test was conducted and we observed that PPD and 5-FU significantly inhibited the cancer cells growth, and the effects of 5-FU were significantly enhanced by the combined treatment with PPD. 5-FU and PPD increased apoptosis, and their co-administration further significantly increased the number of apoptotic cells. Cell cycle experiment data indicated that 5-FU markedly induced the cancer cell S phase arrest while PPD increased the G1 phase arrest. These effects on the arrest at different points in the cell cycle appear to be the pharmacological basis of the observed synergistic effect of these two compounds. Future pharmacokinetic studies will be performed, and the conversion from *in vitro* concentration to *in vivo* dosage will be explored.

In our *in vivo* study, xenografted athymic mice inoculated with HCT-116 human colorectal cancer cells were used. This nude mouse is a strain with a genetic mutation that causes a deteriorated or absent thymus, resulting in the inhibition of the immune system due to a diminished number of T cells. We have previously used this nude mouse model to evaluate the antitumor effects of different natural compounds [40,41]. In this study, in addition to the previously used Xenogen bioluminescence imaging technique, we also compared its results with conventional manual tumor measurements, including both the tumor size (in volume) and tumor weight.

Live animal imaging now is a commonly accepted technique for accurate and quantitative assessment of tumor growth. Bioluminescence imaging systems rely on a bioluminescent signal from tumor cells, generated from the expression of the firefly luciferase gene [42]. However, since adequate tumor blood supply is needed for the bioluminescence imaging result, this technique is not likely suitable for imaging large tumors (usually after Day 20), in which the blood circulation within the tumors may be compromised (unpublished data). Manual tumor size measurement is a traditional way of assessing tumor growth. In addition to being time-consuming, the results of this method could vary among the researchers due to its objective nature. Tumor weight measurement is an accurate approach even when performed by different investigators. However, this method is only applicable when the experimental animals are sacrificed, and thus, quantitatively assessing tumor growth and the effects of therapy over a time course cannot be achieved. To confirm the *in vivo* effects of PPD and 5-FU in nude mice, we used these three methods in our study, and the data obtained suggest that the outcome is consistent.

## 5. Conclusions

In this study, we evaluated the effects of PPD and/or 5-FU on colorectal cancer growth inhibition in both *in vitro* and *in vivo* settings, and obtained consistent results. Compared to the control, 5-FU significantly reduced cancer growth. Our data showed that the co-administration of PPD and 5-FU very significantly inhibited further cancer growth. In combination with 5-FU, PPD enhanced the anticancer action of this commonly used chemotherapeutic agent, and their co-administration may have a clinical value in the management of colorectal cancer.
